# Next-Generation Sequencing and CRISPR/Cas13 Editing in Viroid Research and Molecular Diagnostics

**DOI:** 10.3390/v11020120

**Published:** 2019-01-29

**Authors:** Ahmed Hadidi

**Affiliations:** United States Department of Agriculture, Agricultural Research Service, Beltsville, MD 20705, USA; ahadidi@yahoo.com

**Keywords:** next-generation sequencing (NGS), NGS and viroids, CRISPR-Cas systems, CRISPR-Cas13a system and viroids

## Abstract

Viroid discovery as well as the economic significance of viroids and biological properties are presented. Next-generation sequencing (NGS) technologies combined with informatics have been applied to viroid research and diagnostics for almost a decade. NGS provides highly efficient, rapid, low-cost high-throughput sequencing of viroid genomes and of the 21–24 nt vd-sRNAs generated by the RNA silencing defense of the host. NGS has been utilized in various viroid studies which are presented. The discovery during the last few years that prokaryotes have heritable adaptive immunity mediated through clustered regularly interspaced short palindromic repeats (CRISPR) and CRISPR-associated Cas proteins, have led to transformative advances in molecular biology, notably genome engineering and most recently molecular diagnostics. The potential application of the CRISPR-Cas13a system for engineering viroid interference in plants is suggested by targeting specific motifs of three economically important viroids. The CRISPR-Cas13 system has been utilized recently for the accurate detection of human RNA viruses by visual read out in 90 min or less and by paper-based assay. Multitarget RNA tests by this technology have a good potential for application as a rapid and accurate diagnostic assay for known viroids. The CRISPR/Cas system will work only for known viroids in contrast to NGS, but it should be much faster.

## 1. Introduction

Theodor O. Diener discovered the first viroid in 1971, the causal agent of potato spindle tuber disease [1,2]. He showed that the agent is a free RNA of 25,000–110,000 Daltons, much smaller than a viral genome, and that no viral coat proteins were synthesized in infected plants. He concluded that the RNA is too small to contain the genetic information necessary for self-replication and it must rely on host enzymes for its replication.

Diener’s discovery took place 16 years after another virology landmark discovery in which Heinz Fraenkel-Conrat demonstrated that the genetic information controlling viral replication is carried in the nucleic acid core of each virus particle. He showed that he could actually reconstitute a complete infectious tobacco mosaic virus from the protein and RNA [3].

Viroids, the smallest known infectious agents (246–401 nt), comprise a novel class of infectious single-stranded RNA that replicates autonomously and exists as circular and linear forms with a high degree of base pairing. In contrast to viruses, viroids lack capsid proteins, do not code for proteins and completely dependent on host precellular RNA polymerases and processing enzymes for their replication. 

In contrast to plant RNA and DNA viruses for which many species have been created, viroids currently comprise 32 recognized species and eight putative species [4]. Five and three of the putative species were discovered during the last and current decade, respectively [4]. Viroid species are divided into two families based on the presence or absence of the conserved central region (CCR) in their genome: *Pospiviroidae* (type species, *Potato spindle tuber viroid*) with CCR and members of its five genera replicate and accumulate in host nuclei; *Avsunviroidae* (type species, *Avocado sunblotch viroid*) lacks CCR and has hammerhead ribozymes in both genomic and antigenomic RNAs, and members of its three genera replicate and accumulate in host plastids, mainly chloroplasts [4]. 

Viroids are economically significant as when they infect susceptible plant hosts, they replicate and spread systemically resulting, in most cases, of specific diseases. The damage associated with plant virus infection [5] largely applies to viroids. However, viroid losses, as compared with viruses or other plant pathogens, are generally confined to a country or several countries and do not reach a global level [6]. With the exception of coconut cadang-cadang viroid (CCCVd) and coconut tinangaja viroid (CTiVd) that cause infected coconut palm trees to cease producing coconuts for many years before they die [6], the damage caused by several other viroids in their hosts can be: severe, such as avocado sunblotch viroid (ASBVd) and chrysanthemum stunt viroid (CSVd); moderate, such as potato spindle tuber viroid (PSTVd) and hop stunt viroid (HSVd); mild, such as pome fruit viroids; or variable, such as citrus viroids [6,7,8]. Quantitative data on losses due to viroid infections of susceptible hosts are scarce as only a few yield loss data are available [9]. The yield losses due to infection by PSTVd could range from 17 to 64% [10] and even higher by the third generation of growth [11]. The cone yields of hop plants infected with HSVd in Japan were reduced by 50% or more [12] and infected hop vines could be reduced in height by 35% at the end of the seventh growing season [13]. Infection of coconut palm trees by CCCVd is detrimental as it killed over 40 million coconut palm trees in the Philippines at a cost of four billion US dollars [6]. Citrus bark cracking viroid (CBCVd) infects citrus species with no measurable impact [14], however, when it infects hops it causes an aggressive disease that kills plants in 3–5 years [15,16].

The natural host range of viroids include vegetable and field crops, ornamentals as well as grapevine, fruit trees and palm species. All are members of angiosperms (flowering plants). No viroids have been reported to infect gymnosperms or animals, including primates. Some viroid species such as HSVd and PSTVd have a wide host range while others such as chrysanthemum chlorotic mottle viroid (CChMVd) and coleus blumei viroid (CbVd) 1 to 6 have a narrow one. When a viroid species infects susceptible host, it may cause an economically important disease as discussed above, or it may result in latent (symptomless) infection [7,8,17,18], but they may be pathogenic in other hosts and cause yield reduction and weakness of plants [7,8,17,18,19], depending on the viroid variant nucleotide sequence, host genotype and environmental conditions. Similar to RNA viruses, viroids exist as quasispecies composed of closely related sequence variants with differential properties resulting in heterogeneous progeny. A large number of viroid disease symptoms are similar to those of plant viruses and other pathogens, such as stunting and epinasty of infected plants and different leaf symptoms. Other symptoms may include bark cracking, fruit or tuber malformation, and reduction in number and size of produced flowers. Many factors may affect viroid symptom expression, most notably temperature and host genotype.

The pathogenicity of viroids at the subcellular level may include the formation of paramural bodies or plasmalemmasomes, cell walls and chloroplasts malformations, formation of electron dense deposits in the cytoplasm and chloroplasts. Viroid pathogenicity also involves changes in host metabolism as well as changes in biochemical, molecular and transcriptional mechanisms for viroid disease induction. RNA silencing has also been implicated in viroid pathogenicity. Consequently, single host genes that respond to signals originating from the viroid genome have been identified [7,8].

Viroids are transmitted by mechanical means, grafting, seed, pollen and/or insects [8]. All viroids are transmitted mechanically. With the exception of CCCVd and CTiVd that infect the monocot palm trees, all other viroids are transmitted by grafting. At least 14, seven and six viroids are transmitted by seed, pollen and insect, respectively. Seed-transmitted viroids are: ASBVd; apple scar skin viroid (ASSVd); CbVd; CCCVd; citrus exocortis viroid (CEVd); columnea latent viroid (CLVd); CSVd; eggplant latent viroid (ELVd); HSVd; grapevine yellow speckle viroid-1 (GYSVd-1); pepper chat fruit viroid (PCFVd); PSTVd; tomato apical stunt viroid (TASVd); tomato chlorotic dwarf viroid (TCDVd). Pollen-transmitted viroids include: ASBVd; CCCVd; HSVd; CSVd; PCFVd; peach latent mosaic viroid (PLMVd); PSTVd. Insect-transmitted viroids are: ASBVd; ASSVd; PSTVd; TASVd; TCDVd; tomato planta macho viroid (TPMVd).

The volume and diversity of international exchanges of plant germplasm and newly desired plant cultivars have contributed significantly to international distribution of viroids. Some economically important viroids such as ASSVd, CCCVd, CSVd, HSVd, and PSTVd are transmitted by seed and pollen. The frequency of their occurrence in infected hosts and the harm they cause have created additional pathways for the introduction of these and other viroids in new areas as well as for the emergence of novel viroid variants. It has been shown that the utilization of next-generation sequencing (NGS) in viroid research and diagnosis is sensitive, accurate, and fast using different viroid host plant species, including woody perennial crops such as pome and stone fruits and citrus which have low titers of these pathogens. Moreover, NGS increased the number of novel viroids and variants being discovered and characterized in different plant hosts, including extending the host range of known viroids. Effective genome engineering editing methods, based on clustered regularly interspaced short palindromic repeats (CRISPR) and their associated Cas proteins (CRISPR-Cas), are expected to play a significant role in developing plant resistance to viroid infections as they did for developing transgene-free plants resistant to RNA and DNA plant viruses [20], and have the potential to develop sensitive diagnostic methods for viroids as shown for human viruses [21]. CRISPR/Cas 13 systems have the potential to engineer interference with viroid replication in infected plants as well as to detect viroids in a very short time—about 90 minutes. Thus, both NGS CRISPR/Cas 13 systems have the potential to be used in viroid research and diagnostics as both technologies will be a significant and powerful tool in controlling economically important viroid diseases. In this article we discuss NGS and CRISPR-Cas13 systems as related to viroid research and molecular diagnostics.

## 2. NGS and Viroids

### 2.1. Remarks

NGS platforms became available in the market for the first time in 2000, and their use since 2004 has changed the approach to both basic and applied research in many biological disciplines, including plant virology, which deals with viruses (and their satellites) and viroids. The major advance offered by NGS as compared to first-generation Sanger-based sequencing methods [22] (currently dideoxy chain termination sequencing combined with capillary electrophoresis), is the ability to generate an enormous volume of data, generally in excess of one billion short reads per instrument run, as well as its ability to deliver fast, cost-effective, and accurate genome information [20,23,24,25]. With new NGS platforms continually being developed, the nature of the generated sequence data and the associated costs will likely decrease. Bioinformatics’ software tools are required for NGS data analysis that may include, but are not limited to, alignment of sequence reads, base-calling and/or polymorphism detection, de novo, and genome browsing and annotation. A review of bioinformatics’ software tools available for NGS analysis is beyond the scope of this article but have been the subject of review articles, books as well as journal Bioinformatics. The bioinformatics are continuously being developed and improved to keep up with advances of NGS technologies.

### 2.2. Cost of NGS

**Figure 1:**
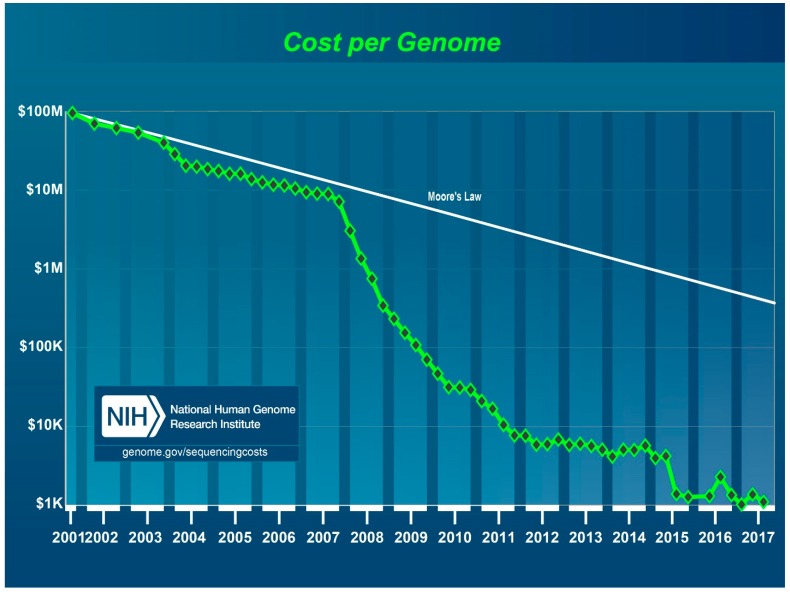
Cost per human genome sequencing in US Dollars from 2001 to 2017 as estimated by the National Human Genome Research Institute, US National Institutes of Health, Bethesda, MD, USA. During this time period, first generation Sanger sequencing methods were used from 2001 through 2007 and NGS platforms from 2008 to 2017.

The estimated costs of NGS of the human genome (3000 Mb) in 2017 are about $ 1000 (Figure 1) [26]. Thus, NGS of viroid genomes (246–401 nt) should be significantly cheaper. 

It is expected in the next few years that the third-generation sequencing (long-read sequencing) platforms will increase sequencing capacity and speed while reducing costs very significantly. Thus, viroid sequencing may be done with costs for less than $ 25–50.

### 2.3. Viroid Studies by NGS 

Because of the small size of viroid genome, its complete genomic sequence can be determined in a single run by NGS as the sequence of hundreds of thousands to millions of viroid-derived small RNAs (vd-sRNA) of 21–24 nt, can be re-assembled to obtain the viroid genome(s) of interest [25]. The vd-sRNA sequences may also be compared to specific sequences of the host genome in order to identify genes that may be down-regulated upon viroid infection vía RNA silencing [8].

NGS of vd-sRNAs, viroid circular and oligomeric RNAs as well as infected host total RNA, DNase-treated total RNA, rRNA-depleted total RNA and rRNA-depleted dsRNA, combined with informatic and computational tools, have been used for almost a decade in viroid research and diagnostics. In most of these studies, NGS data were confirmed by different methods of RT-PCR to ensure that the viroid putative sequence exist in plants. NGS studies may include, but are not limited to, viroid de novo discovery, identification and detection as well as studies of viroid characterization, profiling, distribution, accumulation, biogenesis, strain differentiation, systemic movement, viroid-host interactions, viroid evolution, pathogenesis, mutation, mRNA targeting, extending host range and others as shown in Table 1, Table 2, Table 3, Table 4 and Table 5. 

### 2.4. NGS Identification of Transcriptional (Gene Expression) Changes Associated with Viroid Infection

Methods such as two-dimensional differential gel electrophoresis coupled with mass spectrometry, differential display and microarray have been used to study gene expression in viroid-infected plants [8]. NGS, as compared to these methods, is more sensitive in detecting and identifying transcriptional changes, has higher reproducibility and lower cost. The accurate detailed analyses of transcriptional changes in viroid-infected host are critical for understanding viroid pathogenesis and disease control.

A combination of microarray and large-scale RNA sequence analysis have been used to study gene expression in two PSTVd-infected tomato cultivars: the sensitive cultivar “Rutgers” and the dwarf cultivar “MicroTom” [60]. “Rutgers” infection-related changes were extensive, more than 5, 000 genes with different cellular components were affected. Chloroplast genes were downregulated while many genes encoding proteins associated with nucleus, plasma membrane, ribosomes, cell wall and apoplast were upregulated. It was revealed that “MicroTom” cultivar has a defect in brassinosteroids synthesis and when the hormones were applied exogenously to infected plants, genes involved in stress and other stimuli were upregulated. This observation suggests that potato spindle tuber disease induction may involve brassinosteroid-mediated signaling [60].

NGS was used to identify 11, 600 expressed sequence tags (ESTs) from a cDNA library prepared from total RNA extracted from CSVd-infected chrysanthemum leaves which provided good information of the transcriptome in that system [61]. Approximately 70% of the chrysanthemum ESTs were orthologous to those of five representative plant species [61]. *Orthologous* genes are genes in different species that have similar functions and are similar in their nucleotide sequences which can be traced back to a common ancestral gene. The majority of the identified genes were responsible for diverse metabolic pathways, various stress responses, transcription, translation and transportation as well as genes coding for several cellular components [61].

The gene expression changes in CBCVd in infected hop were revealed by transcriptome analyses [62]. CBCVd infection resulted in extensive modulation of activity of over 200 genes [62]. Expression of genes associated with plant immune responses, hypersensitive responses, phytohormone signaling pathways, photosynthesis, protein metabolism and others were altered. Moreover, genes encoding RNA-dependent RNA polymerase, pathogenesis-related protein, chitinase, as well as those related to defense responses were upregulated.

### 2.5. Searching for and Identifying Ancient Viroids by NGS

For several years, due to advances in NGS technologies, the complete sequence of ancient genomes from both modern and archaic humans has revolutionized our understanding of human evolution and migration [63,64]. NGS analyses of ancient viroids in samples from herbarium, ancient plant samples, soil, air or water may allow us to gain knowledge on the evolutionary history of viroids over the past decades, centuries or even the past few, several or many millennia, as viroids may be “living fossils” of an ancient RNA world [65,66,67].

### 2.6. NGS in Viroid Quarantine and Certification Programs

All known viroids have been sequenced [4,8]. A prior knowledge of viroid sequence is required for RT-PCR detection and identification [68,69,70,71], which is in contrast to detection and identification of known and unknown viroids by NGS [23,24,25]. The cost of RT-PCR-based assay is less expensive than that of NGS analysis, however, the cost of NGS sequencing has become more competitive and affordable during the last couple of years [25]. NGS may be used as the primary diagnostic tool for plant viroids in quarantine and certification programs where various restrictions apply and viroid detection and identification are critical. Viroids of quarantine importance in North America include: ASSVd, PBCVd and PSTVd in Canada; ChCMVd, CSVd, CEVd, CCCVd, ELVd, HSVd (cachexia strain), PLMVd, PBCVd and PSTVd in Mexico; HSVd (cachexia strain), PBCVd and PSTVd in the United States [72]. Viroids of certification importance in propagative material in the European Union include ADFVd, ASSVd, CEVd, HSVd (cachexia strain), PLMVd, PBCVd [72] and CCCVd [73]. Other countries may have similar or different viroids of quarantine or certification importance. For example, all viroids are of quarantine importance in Australia while China considers HSVd (cachexia strain), CEVd and CCCVd of quarantine importance, and Chile considers ASBVd, CCCVd, PSTVd, TASVd and TCDVd of quarantine importance [72].

NGS revealed in 2015 that the causal agent of severe stunting and death of hop plants in Slovenia is CBCVd [16]. As a result, the European and Mediterranean Plant Protection Organization (EPPO) added the viroid to “The EPPO Alert List” [16], suggesting that NGS technology could/should be adopted as a certification or post-entry quarantine measure to detect and identify CBCVd in hops. NGS may become instrumental in releasing plants in quarantine and certification programs at a faster rate than current strategies while improving our ability to prevent the introduction of foreign viroids into new countries (this article, [48]).

## 3. Potential Utilization of CRISPR-Cas 13 Systems in Viroid Interference and Diagnostics

### 3.1. General Aspects

Clustered regularly interspaced short palindromic repeats (CRISPR) and CRISPR-associated Cas proteins comprise the CRISPR-Cas systems, which confer adaptive immunity against foreign genetic elements such as bacteriophages and plasmids in many bacteria and most archaea [74,75,76,77]. CRISPR-Cas systems act as RNA-guided programmable nucleases to degrade DNA and/or RNA derived from foreign nucleic acids by preserving molecular memory information of prior infections [78,79,80,81,82]. Three processes are involved in CRISPR/Cas-mediated immunity: adaptation, transcription and processing, and interference (for review, see Ref. [77,81]). Adaptation includes information of the most recent infection during which “spacer” sequences (short segments of foreign DNA) acquired from the invader genome, such as plasmids and bacteriophages [74,83,84], are integrated into the prokaryotic genome [78]. That followed by placing the spacer sequences into a CRISPR array in between pairs of short repeated sequences (for array review see Ref. [81]). The array is then transcribed to generate pre-crRNAs, which are further processed to generate the mature crRNAs. Thus, the spacer sequences provide the sequence specificity for interfering with invading DNA and/ or RNA. Interference, whereby CRISPR Cas enzymes are guided by the crRNAs to form “effector or interference complexes” that enables crRNAs to target, cleave and degrade complementary foreign nucleic acids in the respective invader genomes, thus preventing further infection [74,75,77,78,79,80,81,82,83,84,85,86,87,88,89].

The CRISPR-Cas systems are divided into two general classes on the basis of the composition of the interference complex [82]. Class 1 systems rely on multi-effector complexes mediate the interference whereas Class 2 systems utilize single multi-domain effectors to mediate the interference. These two classes are further divided into six types and 33 subtypes based on the genomic composition of the CRISPR array and the signature interference effector. Class 1 includes types I, III, and IV, and Class 2 includes types II, V and VI [82,86,87]. Types I, II and V target double-stranded DNA but types III and VI target single-stranded RNA [82,88,89,90,91].

Class 2 type VI systems, include a single “effector” protein designated Cas13a, formerly C2c2 [91], which when combined with crRNA forms a crRNA-guided RNA-targeting CRISPR effector complex [82,91,92]. Cas13a provides specificity through CRISPR RNA (crRNA)-target pairing and additional sensitivity due to signal amplification by Cas13a collateral cleavage activity [93]. Cas13a possesses two enzymatically distinct ribonucleases activities that are needed for optimum interference [92]. The first RNase is required for pre-crRNA processing to help formation of mature interference complexes while the second RNase, which is provided by the two Higher Eukaryotes and Prokaryotes Nucleotide-binding (HEPN) domains [94], is required for degradation of target RNA during viral interference. The RNase activity provided by HEPN in Cas13a is lacking in other known Cas proteins [95].

Classical breeding and different molecular strategies to introduce resistance to viroids have been unsuccessful or partially successful in containing viroid infection [96]. A new molecular approach for controlling viroid infection by CRISPR-Cas13a system has the potential to be useful and successful. This system has been recently utilized successfully to engineer interference with an RNA plant virus, turnip mosaic virus, in *Nicotiana benthamiana* [95].

### 3.2. Application to Target Viroids for Inactivation

PSTVd, PLMVd and ASBVd are economically important and their genomes and replication have been studied extensively [8]. Selected motifs of each of the above viroids [97] could be specifically targeted by CRISPR Cas13a system. Briefly, the following viroid motifs could be targeted (as indicated in [97]):

“A-PSTVd terminal left domain: 1-Initiation site of minus PSTVd RNA synthesis at nt U359 or C1. 2-Three bulges associated with plus PSTVd RNA replication.

B-PSTVd center conserved region: 1- Hairpin I, delimited by nt 79 and 110 on the upper strand of the viroid RNA. 2- Hairpin II, delimited by nt 227 and 328 on the lower strand of the viroid RNA. 3-Loop E, located between nt 5’-G97 to C103-3’ and nt 5’-G255 to C262-3’.

C-PSTVd terminal right domain: RY motif, composed of two asymmetric internal loops, with sequence elements 5’-ACAGG-3’ (nt 173-177) in the upper strand and 3’-CUCUUCC-5’ (nt 190–184) in the lower strand.

D-PLMVd initiation sites: Such sites are located at nt C51 for the plus strand and at nt U286 for the minus strand, both mapping at similar double-stranded motifs of 6–7 bp that also contain the highly conserved GUC triplet preceding the self-cleavage site in both polarity strands. These motifs are located at the base of a similar long hairpin that presumably contains the promoters for a chloroplastic RNA polymerase. Since the in vivo initiation of PLMVd RNAs occurs near the self-cleavage (and self-ligation) sites, CRISPR-Cas13a editing in this region would attenuate both the transcription and processing of PLMVd RNA.

E-ASBVd initiation sites: These sites are located at nt U121 and at nt U119 for the plus and minus strands, respectively. The initiation sites are only two nucleotides apart and each site starts with the same sequence, UAAAA, which suggests that the viroid promoters are formed, at least in part, by the sequences flanking the two initiation sites.”

### 3.3. Application to Viroid Detection

The RNA guided nuclease activities of CRISPR-Cas13a, -Cas13b and -Cas12a, which are functionally distinct from -Cas9, have been recently utilized to develop sensitive detection methods for human viruses [98,99,100,101]. CRISPR-Cas13a and -Cas 13b were used for the detection of RNA viruses [98,99,100] and -Cas12a for DNA viruses [99,101].

It has been shown that the Cas13 SHERLOCK (specific high-sensitivity enzymatic reporter unlocking) platform can incorporate pre-amplified input material to develop a paper-based assay with improved sensitivity [98]. SHERLOCK conflates recombinase polymerase amplification (RPA) [102] with highly specific Cas13-based detection. Cas13a [91] and Cas13b [103,104] homologs have discrete crRNA and substrate preferences to simultaneously detect multiple transcripts [99,100,105]. When Cas13 was combined with Csm6, an auxiliary CRISPR-type III associated nuclease [106], an increase of signal sensitivity by approximately 3.5-fold was observed [99]. The revised platform, termed SHERLOCKv2, can simultaneously detect three ssRNA targets and one DNA target in a single reaction [99]. It is accurate and quantitative. By characterization of 17 CRISPR-Cas13a and -Cas13b enzymes and selecting three with distinct cleavage preferences and combining them with a Cas12a enzyme and RPA, synthetic ssRNA, dengue virus ssRNA, Zika virus ssRNA and synthetic dsDNA were accurately detected by a visual readout in less than 90 minutes [99] and in a readily deployable format via a paper-based assay [100].These results highlight the potential of such an approach as a multiplex, rapid and quantitative detection assay for viroid RNA. The CRISPR/Cas system will work only for known viroids, in contrast to NGS, but it should be much faster.

## 4. Final Remarks

CRISPR-Cas editing does not incorporate permanently foreign DNA into the host genome as in transgenic plants, and agricultural applications of this precise technology are already creating valuable products for various markets in the United States. For this reason, the U.S. Department of Agriculture does not regulate foods or plants developed by CRISPR-Cas editing [107,108,109]. Thus, this technology can be used in the US to develop plants resistant to viroids or other pathogens. In the European Union (EU), however, the highest court ruled on July 25, 2018 that CRISPR-Cas-edited plants are subject to the same regulations as those of conventional genetically modified organisms [110], which were imposed in EU about two decades ago. Consequently, CRISPR-Cas-edited plants need to go through a lengthy approval process.

## Figures and Tables

**Table 1:** Discovery of novel viroids or variants by NGS.

Viroid	Target RNA	Remarks	Reference
Persimmon viroid-2 (PVd-2)	dsRNA	A novel apscaviroid	[27]
Grapevine latent viroid (GLVd)	total RNA	A novel apscaviroid	[28]
Apple dimple fruit viroid (ADFVd)	vd-sRNAs	A novel variant that naturally infects fig	[29]
CBCVd	vd-sRNAs or total RNA	A novel variant that naturally infects hops	[16]
	rRNA-depleted libraries	Two novel citrus variants closely related to hop variants	[30]

**Table 2:** Extending the host range of viroids by NGS.

Viroid	Target RNA	Remarks	Reference
ADFVd	vd-sRNAs	The host range was naturally extended to fig trees	[29]
CBCVd	vd-sRNAs or total RNA	The host range was naturally extended to cultivated hops	[16]
Apple fruit crinkle viroid (AFCVd)	vd-sRNAs	The host range was extended to tomato, cucumber and wild hop using an inoculum of a variant from cultivated hop	[31]
HSVd	vd-sRNAs	Extending the host range to chickpea	[32]

**Table 3:** Viroid mutants and quasi-species identified by NGS.

Viroid	Target RNA	Remarks	Reference
PLMVd	DNase-treated total RNA	A single infecting variant mutates quickly (about 17% variation compared to the parental sequence)	[33]
PSTVd	vd-sRNAs	Different variants used to elucidate the viroid quasispecies evolved during infection. Several novel and already known variants were competent in replication. Common strand-specific mutations identified	[34]
	vd-sRNAs	Plus and minus vd-sRNAs of three different viroid variants and their mutants in tomato were identified and analyzed	[35]
PSTVd and ELVd	viroid circular and oligomeric RNAs	Chloroplastic and nuclear viroids have different mutation rates	[36]

**Table 4:** vd-sRNAs: characterization, biogenesis pathway and target host mRNAs identified by NGS.

Viroid	Remarks	Reference
GYSVd-1 and HSVd	Biogenesis and role of vd-sRNAs of the two viroids in grapevine plant-viroid interactions	[37]
GYSVd-1, GYSVd-2 and HSVd	vd-sRNAs of these grapevine viroids were characterized	[38]
HSVd	Study the pathway involved in the biogenesis of vd-sRNAs in cucumber	[39]
PLMVd	Study the viroid vd-sRNAs genesis, pathogenesis and evolution	[40,41]
	vd-sRNAs containing the pathogenic determinant of the viroid guide degradation of a host mRNA as predicted by RNA silencing, thus leading to symptom expression	[41]
PSTVd	RNA-dependent RNA polymerase 6 of *Nicotiana benthamiana* restricts accumulation and precludes meristem invasion of the viroid, which replicates in nuclei with prevailing 21-22 nt plus-strand vd-sRNAs that adopt strand-specific hot spot profiles	[42]
	vd-sRNAs derived from the virulence modulating region of two viroid variants target callose synthase mRNAs, which may affect the viroid spread/accumulation and symptom severity in tomato	[43]
	vdsRNAs, originated from viroid variants that induce different symptoms, may target different host mRNAs	[44,45]
	Study possible interactions of vd-sRNAs of two variants of the viroid plus and minus strands with host mRNAs during infection. vd- sRNAs induction was found independent of host mRNAs degradation	[46]
	Considering that vd-sRNAs of 21-24 nt are generated in infected plants, bioinformatic tools were utilized to detect and identify viroids and viroid-like circular RNAs in sRNA libraries	[28,47]

**Table 5:** Detection and identification of viroids in host plants by NGS.

Viroid	Target RNA	Host	Reference
ADFVd	vd-sRNAs	apple, fig	[29,48]
	dsRNA	apple	[48]
AFCVd	dsRNA	apple	[48]
ASSVd	Total RNA or dsRNA	apple	[28,48]
Australian grapevine viroid (AGVd)	Total RNA or dsRNA	grapevine	[49]
CEVd	DNase-treated total RNA	grapevine	[50]
CBCVd	vd-sRNAs or total RNA	hop	[16]
CLVd	vd-sRNAs or total RNA depleted of rRNA	tomato	[51]
GLVd	Total RNA	grapevine	[28]
GYSVd-1	vd-sRNAs vd-sRNAs or dsRNA	grapevine	[37,38,52,53] [54]
	DNase-treated total RNA		[50]
	Total RNA		[55,56]
GYSVd-2	vd-sRNAs	grapevine	[38]
HSVd	vd-sRNAs	Chickpea Grapevine cucumber	[32] [37,38,52,53] [39]
	DNase-treated total RNA	grapevine	[50]
	dsRNA total RNA	*Prunus* sp. grapevine	[48] [56]
PLMVd	vd-sRNAs	*Prunus* sp.	[33,40,57]
	vd-sRNAs or total RNA depleted of rRNA dsRNA	*Prunus* sp. *Prunus* sp.	[51] [48]
Pear blister canker viroid (PBCVd)	dsRNA	pear	[48]
PVd-2	dsRNA	persimmon	[27]
PSTVd	vd-sRNAs	tomato	[42,43,44,58,59]
TASVd	vd-sRNAs or total RNA depleted of rRNA	tomato	[51]

## Dedication

This article is dedicated to the memory of Dr. Heinz Fraenkel-Conrat with whom the author was NIH Postdoctoral Fellow from 1970 to 1973 at the Department of Molecular Biology, University of California, Berkeley.
